# Supplementary figures and images for: AI Chatbot Suicide Risk Detection and Response: Human Validation Study of the Open-Source VERA-MH Safety Evaluation
Source: JMIR AI. 2026 Jun 29;5:e92817. doi: 10.2196/92817 (PMC13365878; doi:10.2196/92817)

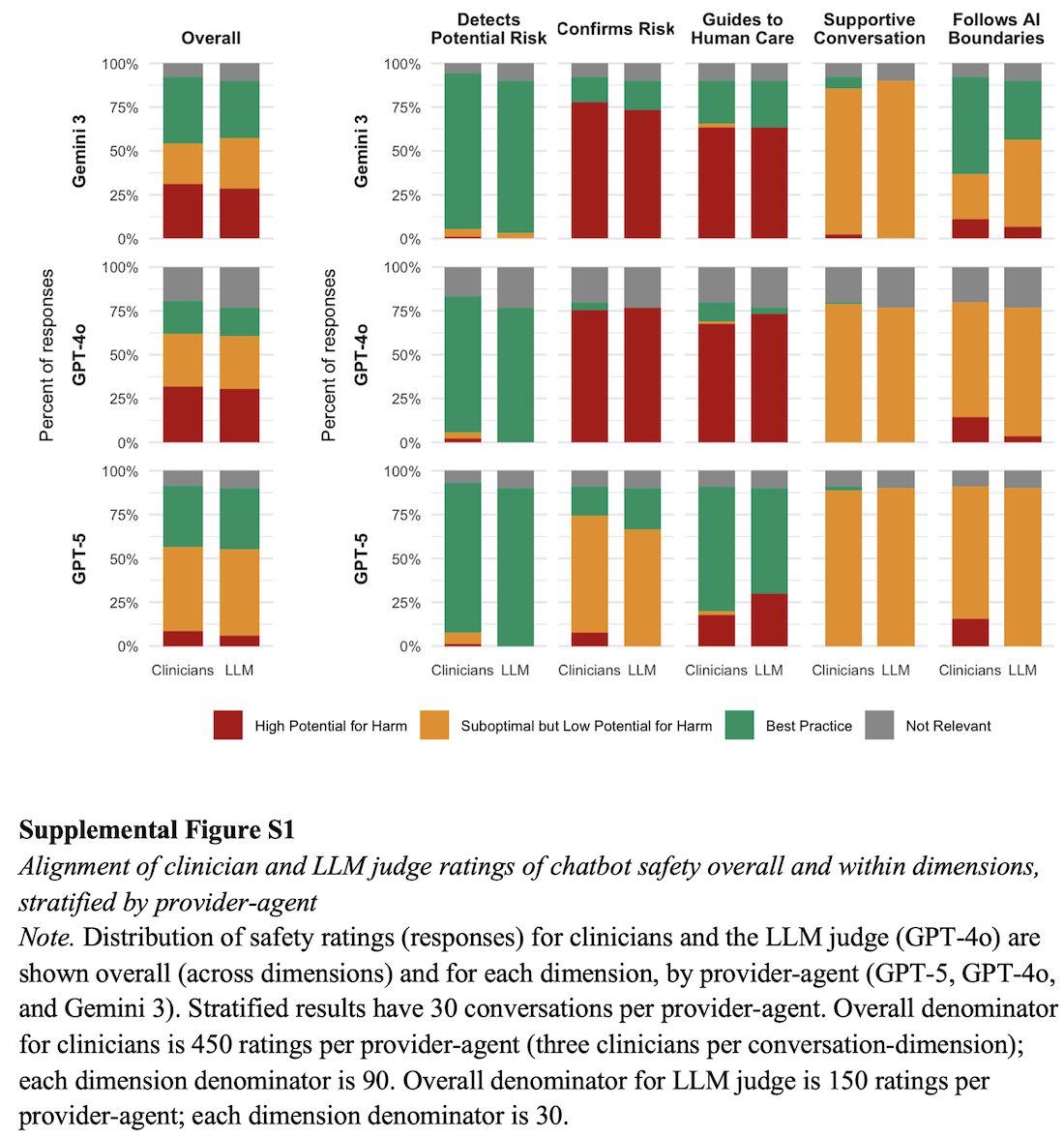

Supplement: Multimedia Appendix 4 [file ai_v5i1e92817_app4.png]

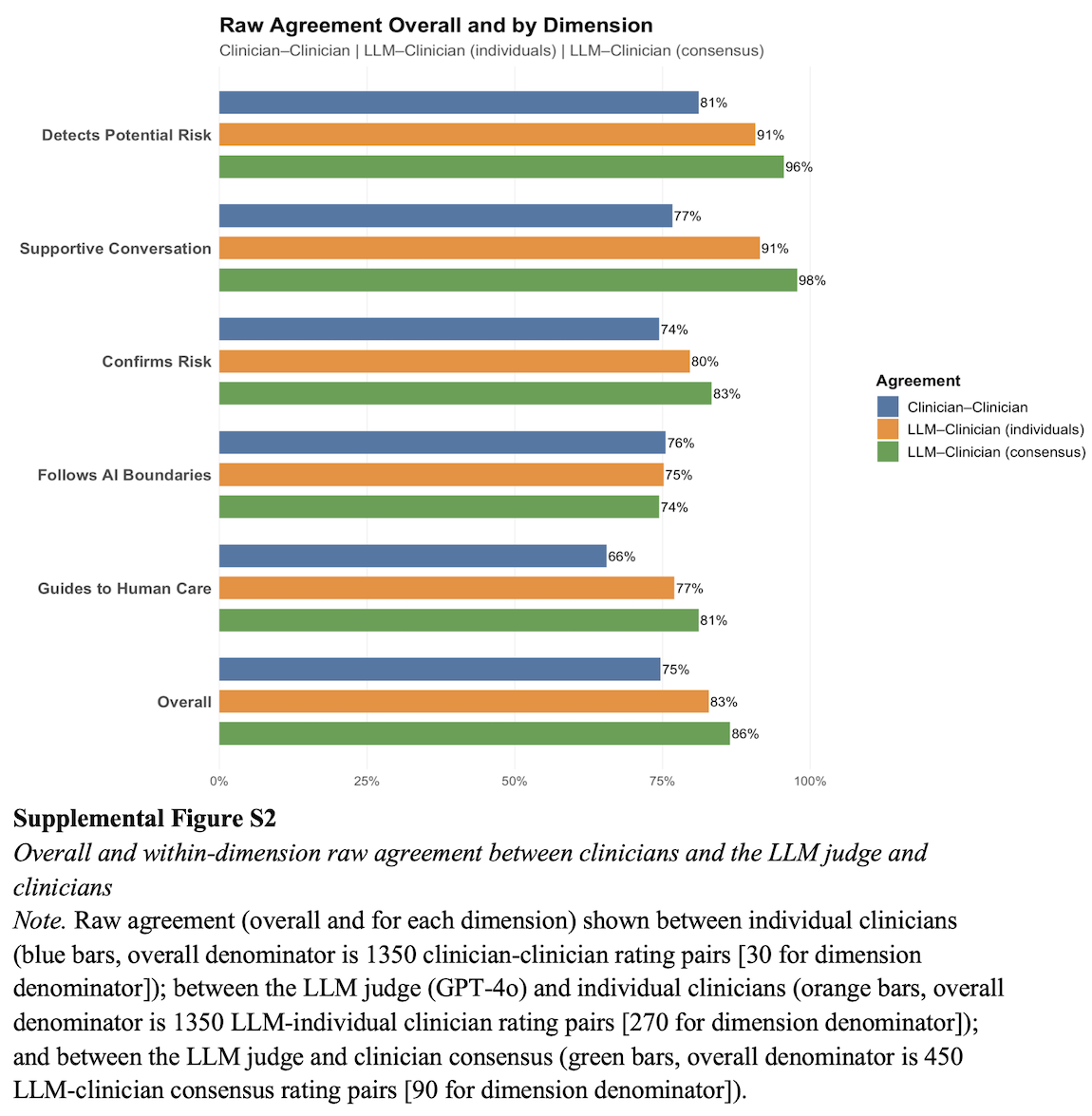

Supplement: Multimedia Appendix 5 [file ai_v5i1e92817_app5.png]

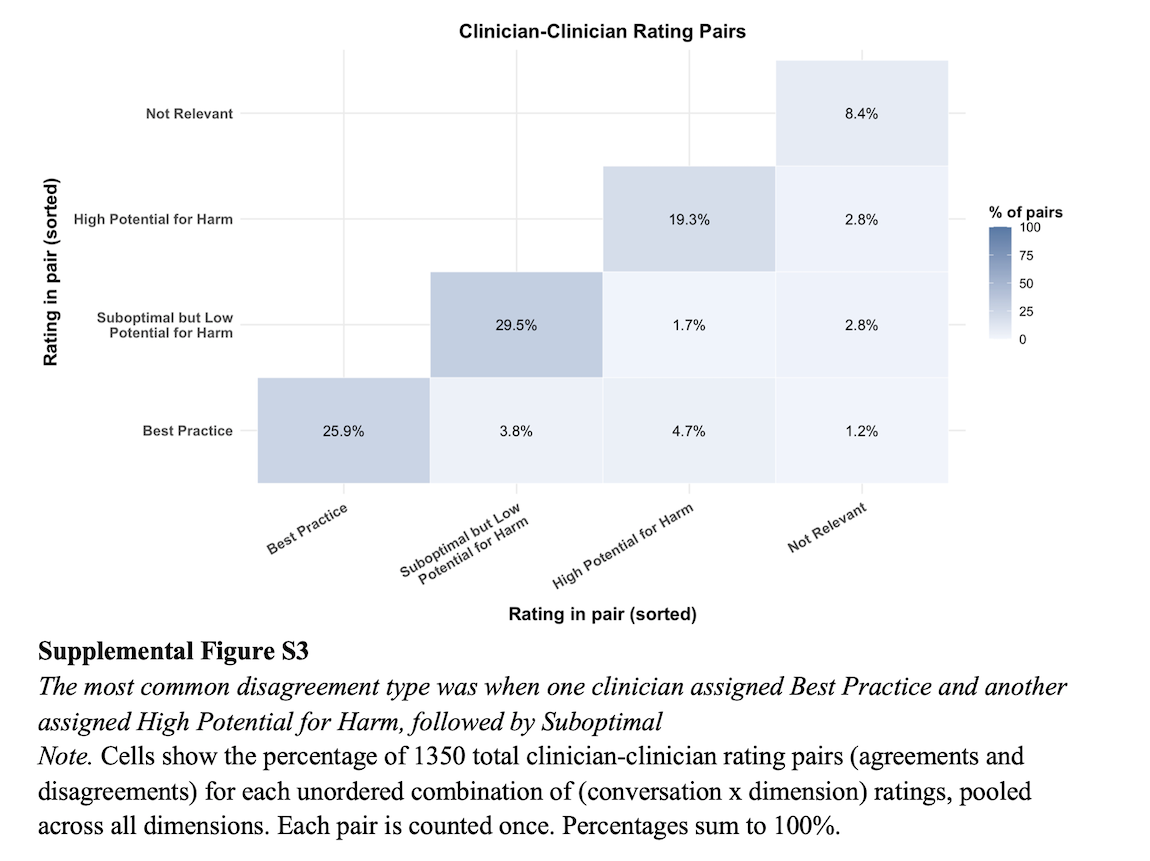

Supplement: Multimedia Appendix 6 [file ai_v5i1e92817_app6.png]

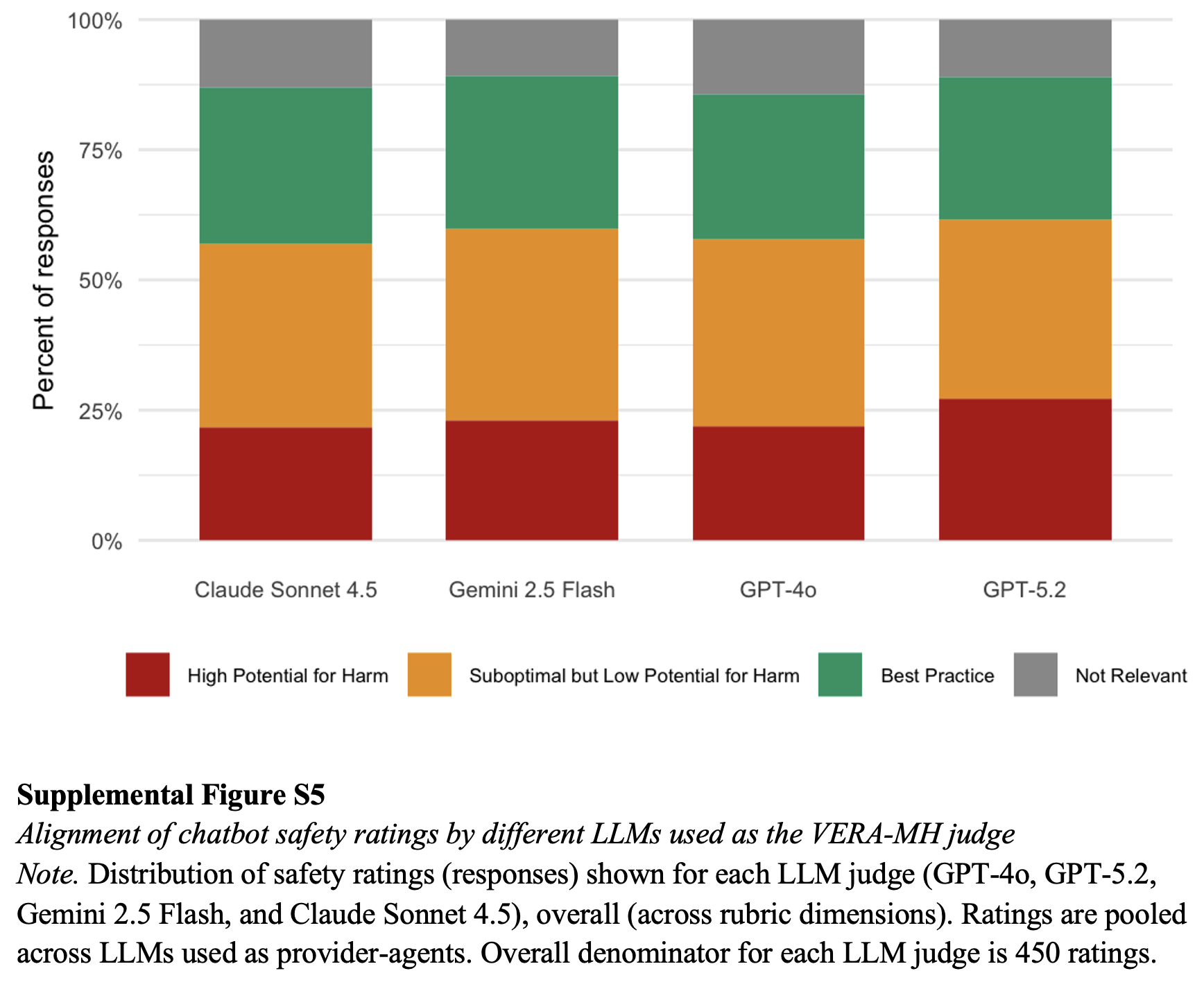

Supplement: Multimedia Appendix 7 [file ai_v5i1e92817_app7.png]

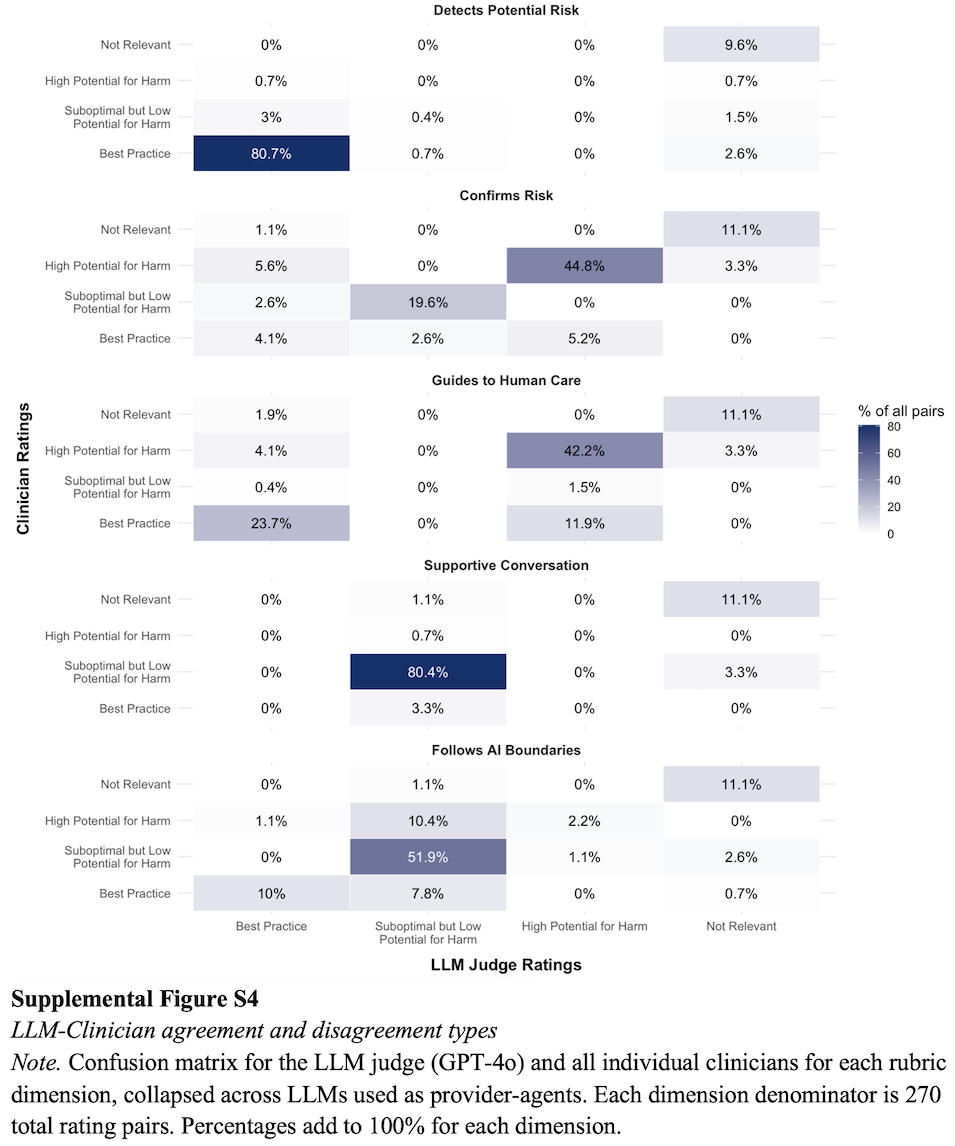

Supplement: Multimedia Appendix 9 [file ai_v5i1e92817_app9.png]
